# The clinical effectiveness of wound edge protectors in reducing surgical site infection after abdominal surgery: meta-analysis

**DOI:** 10.1093/bjsopen/zrac065

**Published:** 2022-05-11

**Authors:** Xujia Li, Haomin Lin, Lin Zhu, Jing Chen, Sujuan Lei, Bo Li, Song Su

**Affiliations:** Department of General Surgery (Hepatobiliary Surgery), The Affiliated Hospital of Southwest Medical University, Luzhou, Sichuan, China; Department of General Surgery (Hepatobiliary Surgery), The Affiliated Hospital of Southwest Medical University, Luzhou, Sichuan, China; Department of General Surgery (Hepatobiliary Surgery), The Affiliated Hospital of Southwest Medical University, Luzhou, Sichuan, China; Department of General Surgery (Hepatobiliary Surgery), The Affiliated Hospital of Southwest Medical University, Luzhou, Sichuan, China; Department of General Surgery (Hepatobiliary Surgery), The Affiliated Hospital of Southwest Medical University, Luzhou, Sichuan, China; Department of General Surgery (Hepatobiliary Surgery), The Affiliated Hospital of Southwest Medical University, Luzhou, Sichuan, China; Department of General Surgery (Hepatobiliary Surgery), The Affiliated Hospital of Southwest Medical University, Luzhou, Sichuan, China

## Abstract

**Background:**

Surgical site infection (SSI) is a common complication after abdominal surgery. The effectiveness of wound edge protectors in reducing infection of the surgical sites is still unclear. The purpose of this study was to determine the clinical effectiveness of a wound edge protector (WEP) in reducing SSI rates after abdominal surgery.

**Methods:**

PubMed, Embase, Web of Science, and the Cochrane Library were systematically searched to obtain relevant articles published up to September 2021. Publications were retrieved if they contain primary data on the use of WEPs in reducing SSI compared with standard care in patients undergoing abdominal surgery. Subgroup analyses were performed for different WEP types, surgical sites, and levels of contamination. The outcome of interest was a clinically defined SSI. Qualitative variables were pooled using risk ratios (RRs).

**Results:**

Twenty-two eligible randomized clinical trials involving 4492 patients were included in this meta-analysis. WEP was associated with the reduced incidence of overall SSI (RR = 0.66; 95 per cent c.i. 0.53 to 0.83; *P* = 0.0003), and superficial SSI (RR = 0.59; 95 per cent c.i. 0.38 to 0.91; *P* = 0.02). In addition, WEP also successfully reduced the risk of SSI in clean-contaminated wounds (RR = 0.61; 95 per cent c.i. 0.40 to 0.93; *P* = 0.02) as well as in contaminated wounds (RR = 0.47; 95 per cent c.i. 0.33 to 0.67; *P* < 0.0001); however, WEP did not reduce SSI incidence in colorectal surgery (RR = 0.68; 95 per cent c.i. 0.46 to 1.01; *P* = 0.05).

**Conclusion:**

This study suggests that WEP was efficient in reducing superficial SSI. Both double-ringed and single-ringed devices were efficient in reducing SSI. WEP was effective in reducing SSI incidence in clean-contaminated and contaminated surgery; however, its use does not reduce the SSI rate in colorectal surgery.

## Teaser

In this study, we included four newly published randomized clinical trials, involving more than 500 participants, to demonstrate the clinical efficacy of wound edge protectors (WEPs) based on reliable evidence. In addition, for the first time, we performed a subgroup analysis examining different surgical sites. Our results suggested that the use of WEP does not reduce the SSI rate in colorectal surgery.

## Introduction

Surgical site infection (SSI), as defined by the Centers for Disease Control and Prevention (CDC), is an infection that occurs in the operative site within 30 days of surgery, or within 1 year after placing an implant^1–3^. SSIs can be divided into different categories based on location, with a differentiation for skin and subcutaneous tissue (superficial incisional), deep soft tissue (deep incisional), and other anatomical sites (organ/space)^4^. Bacterial colonization on a patient’s skin, alimentary canal, and genital tract are the most common sources of SSIs^5^. Compared with other types of surgeries, abdominal surgeries are typically performed with a clean-contaminated or contaminated incisional wound and are related to higher SSI rates ranging between 15 and 25 per cent^6–8^. SSI is one of the most common complications of abdominal surgery, and it significantly increases postoperative disability rate, mortality, and medical expenses^9–11^. Therefore, it is essential to reduce the incidence of SSI to improve patients’ quality of life as well as decrease medical costs.

For more than 50 years, wound edge protectors (WEPs), both single- and double-ringed, have been frequently used to protect the incisional wound and reduce SSI rates in patients undergoing surgery^12^. As endogenous pathogens from the skin and gastrointestinal tract are the major contributors of postoperative SSIs after abdominal surgery^13^, physical WEPs can markedly reduce SSI rates by protecting the incision from sources of infection, such as intestinal content spillage. While WEPs effectively protect wound edges from bacterial invasion, the effectiveness of WEPs on reducing the SSI rate in abdominal surgery remains unclear^14^. Despite the expected clinical benefit of WEPs in reducing SSI rate and the actual WEP benefit reported in several studies^12,15–22^, some randomized clinical trials (RCTs) still reported disappointing conclusions of WEPs usage in reducing SSI rates in patients undergoing abdominal surgery^23–31^. In addition, previous systematic reviews, based on limited numbers of patients, revealed multiple limitations in the WEPs and, therefore, called for high-quality multicenter RCTs^32–37^. Since then, several high-quality trials, involving more than 500 participants, have been published, necessitating our current reassessment and meta-analysis, which will illustrate the clinical effectiveness of WEPs, based on reliable evidence^38–41^.

The purpose of this review was to assess the efficacy of WEPs in preventing SSI in patients undergoing different abdominal surgical procedures with different levels of contamination.

## Methods

### Search strategy

The literature was searched systematically using PubMed, Embase, Web of Science, and Cochrane Library for studies published up to 30 September 2021. This systematic search was conducted according to the PRISMA guidelines^42^. The Medical Subjects Heading terms were used in combination with Boolean operators AND or OR: ‘surgical site infection’ and ‘wound edge protection device.’ Equivalent free-text search terms such as ‘wound infection’, ‘deep wound infection’, ‘deep space infection’, and ‘postoperative surgical infection’ are used in combination with ‘wound edge protector device’, ‘wound edge protector’, ‘wound protector’, ‘circular wound protector and retractor’, ‘surgical wound isolator’, ‘Alexis’, and ‘surgical infection protector’. The titles from the search results were examined and determined to be suitable for potential inclusion in the study. Furthermore, the references in relevant articles were also screened manually to identify additional eligible studies. The language or time was not restricted.

### Selection criteria

The literature was searched for randomized clinical studies that were included if they met all the following criteria: a study comparing the use of WEP and the non-use of WEP in abdominal surgery; patients undergoing abdominal surgery; and main outcome measures reported preferably as an intention-to-treat analysis. For data from repeated studies or shared across multiple studies, the analysis will only include data from the first published paper. Articles published in the form of a retrospective study, prospective non-randomized study, animal study, review, letter, meeting, or comment were excluded.

### Study selection and data extraction

The title and abstracts of the search results were scanned for potentially eligible studies. Then, the full texts of potentially eligible studies were screened to determine whether they should be included based on the inclusion criteria. The authors were contacted via e-mail to obtain any missing information. Data from the included articles were extracted independently by three reviewers, and inconsistencies were resolved by consensus. After completing the data extraction, the two independent reviewers discussed the results and, if discrepancies were present, a consensus was reached before analysis.

### Methodological quality assessment

The methodological quality for the included studies was assessed independently by three researchers. The quality of RCTs was assessed by the Cochrane risk of bias tool. The included trials were graded as high quality, moderate quality, or based on the following criteria: trials were considered as high quality when both randomization and allocation concealment was assessed as low risk of bias, and all other items were assessed as low or unclear risk of bias in a trial; trials were considered moderate quality if they did not meet the criteria for high or low risk; and trials were considered low quality if either randomization or allocation concealment was assessed as a high risk of bias, regardless of the risk of other items.

### Outcomes of interest

The outcome of interest was dichotomous: presence or absence of an SSI. The main analysis examined the efficacy of WEPs in reducing the rate of SSI after abdominal surgery. In addition, the following pre-specified subgroup analyses (effectiveness of WEP *versus* non-WEP on SSI rate) were performed: for different types of WEPs (single-ring and double-ring); for different abdominal surgical sites (colorectal, upper digestive tract/small intestine, hepatobiliary and pancreatic, and appendix); and for different degrees of intraoperative contamination (clean, clean-contaminated, contaminated, or dirty operations as defined by the CDC^13^).

### Statistical analysis

Statistical analyses were performed with the recommendations of the Cochrane Collaboration guidelines. The meta-analysis included the use of Review Manager 5 Software (the Cochrane Collaboration, Oxford, UK), random-effects model analysis, heterogeneity testing by chi-squared test, heterogeneity quantification by *I*^2^ test, and the use of forest plots for the graphical display of the combined outcomes. Dichotomous variables were presented as risk ratio (RR) with 95 per cent confidence interval (c.i.). Sensitivity analyses were performed by reanalysis of the data after removing each trail to assess the robustness of polled results.

## Results

**Fig. 1:**
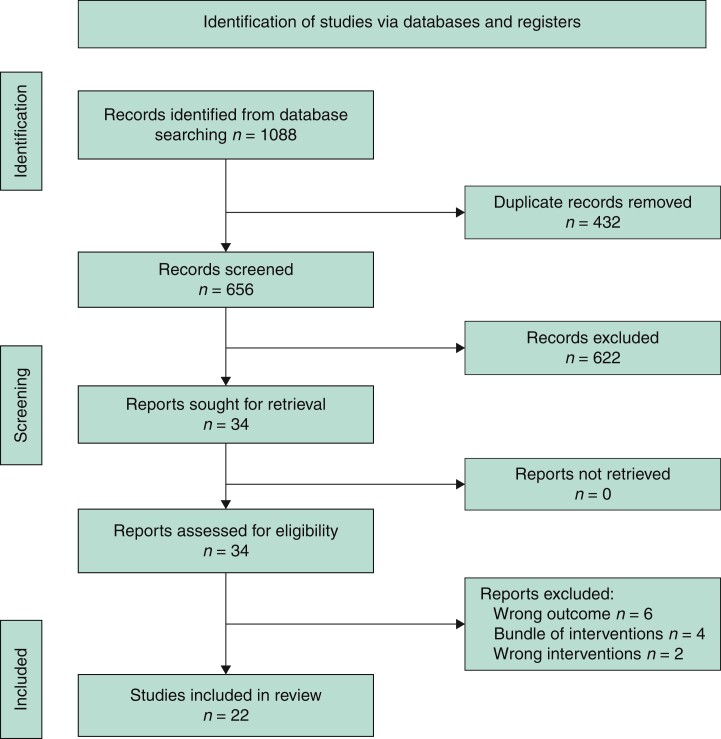
PRISMA flow diagram of literature search and selections

Initially, 1088 articles were obtained through a search of PubMed (*n* = 401), Embase (*n* = 451), Web of Science (*n* = 183), and the Cochrane Library (*n* = 53). After reviewing the titles and abstracts, 403 duplicates, and 622 irrelevant articles were excluded. Full texts of the remaining 34 potentially eligible articles were screened for assessment. Twenty-two studies were finally included in this meta-analysis (*Fig. 1*).

Information on the included studies, such as author, year of publication, country, study design, number of patients, and major outcomes are listed in *Table 1*.

**Table 1:** General characteristics of studies in the final analysis

No	Study	Surgical sites	Ring type	Sample size (intervention *versus* control)	Conclusion (efficacy of WEPs)
**1**	Maxwell, 1969^12^	Abdominal surgeries	Single	120 (88 *versus* 32)	Yes
**2**	Williams, 1972^29^	Abdominal surgeries	Single	167 (84 *versus* 83)	None
**3**	Psaila, 1977^28^	Abdominal surgeries	Single	93 (46 *versus* 47)	None
**4**	Nyström, 1980^22^	Appendicectomy	Single	275 (132 *versus* 143)	
**5**	Gamble, 1984^26^	Colorectal resection	Single	56 (27 *versus* 29)	None
**6**	Nyström, 1984^27^	Colorectal resection	Single	140 (70 *versus* 70)	None
**7**	Batz, 1987^31^	Colorectal resection	Single	50 (25 *versus* 25)	None
**8**	Sookhai, 1999^21^	Abdominal surgeries	Single	352 (170 *versus* 182)	Yes
**9**	Ozer, 2006^30^	Appendicectomy	Dual	122(64 *versus* 58)	None
**10**	Horiuchi, 2007^18^	Gastrointestinal surgeries	Dual	221 (111 *versus* 110)	Yes
**11**	Silva, 2008^19^	Appendicectomy	Dual	433 (221 *versus* 212)	Yes
**12**	Lee, 2009^20^	Appendicectomy	Dual	109 (61 *versus* 48)	Yes
**13**	Reid, 2010^17^	Colorectal resection	Dual	130 (64 *versus* 66)	Yes
**14**	Baier, 2012^24^	Colorectal resection	Single	199 (98 *versus* 101)	None
**15**	Cheng, 2012^16^	Colorectal resection	Dual	64 (34 *versus* 30)	Yes
**16**	Lauscher, 2012^25^	Colorectal resection	Single	93 (46 *versus* 47)	None
**17**	Pinkney, 2013^23^	Abdominal surgeries	Single	735 (396 *versus* 366)	None
**18**	Mihaljevic, 2014^15^	Abdominal surgeries	Single	546 (274 *versus* 272)	Yes
**19**	Bressan, 2018^38^	Pancreaticoduodenectomy	Dual	107 (57 *versus* 50)	Yes
**20**	Kobayashi, 2019^39^	Colorectal resection	Single	100 (50 *versus* 50)	Yes
**21**	De Pastena, 2020^41^	Pancreaticoduodenectomy	Dual	190 (94 *versus* 96)	None
**22**	Muniandy, 2021^40^	Appendicectomy	Dual	190 (95 *versus* 95)	None

WEP, wound edge protector.

**Fig. 2:**
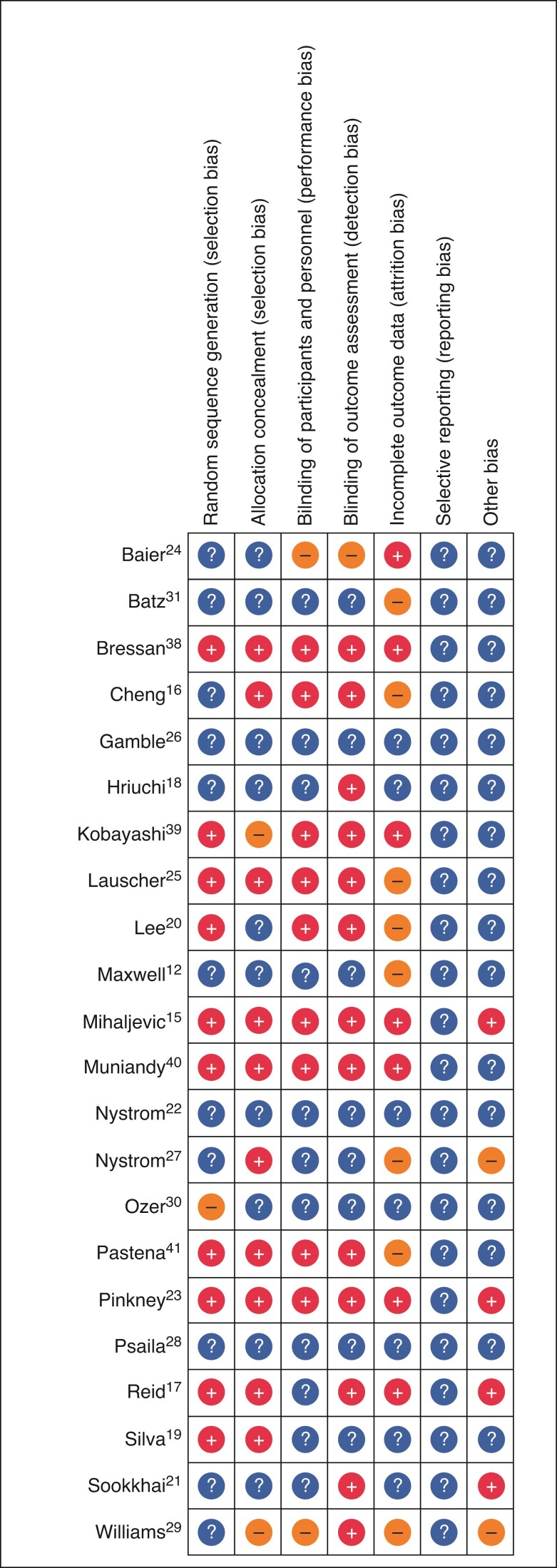
Risk of bias of included studies

The risk of bias in the 22 RCTs was evaluated by the Cochrane risk of bias tool. The results of the quality assessment for the 22 included studies are shown in (*Fig. 2*). Six included trials were considered as high quality^15,17,19,23,38,40^, 13 were considered moderate quality^12,16,18,20–22,24–28,31,41^, and 2 were considered low quality^29,30,39^ (*Fig. 2*).

+indicates low risk of bias; −indicates high risk of bias;indicates unclear risk of bias.

**Fig. 3:**
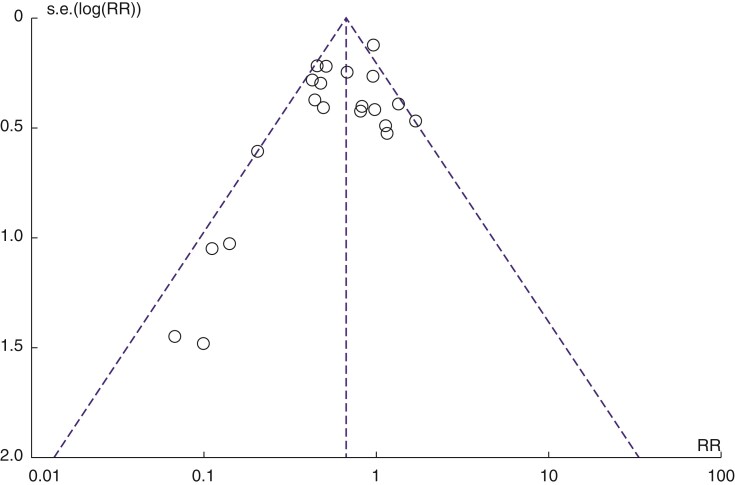
Funnel plot of the included RCTs comparing WEPs with control (RevMan 5.3 output)

RCT, randomized clinical trial; WEP, wound edge protector; RR, risk ratio; M-H, Mantel-Haenszel.

The risk of publication bias was examined using a funnel plot (*Fig. 3*). The asymmetry of this graph is caused by four studies^16,20,30,31^.

### Analysis of pooled data

**Fig. 4:**
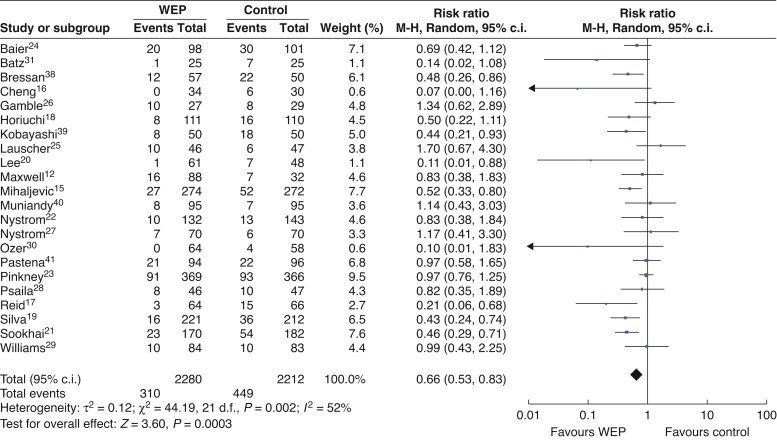
Forest plots for overall rate of surgical site infection

WEP, wound edge protector; M-H, Mantel-Haenszel.

The individual RRs and 95 per cent confidence intervals for the random-effects model meta-analysis of included RCTs are shown in (*Fig. 4*). In the meta-analysis of 4492 patients from 22 RCTs, WEP significantly reduced the incidence of SSI. Moreover, the difference was statistically significant (RR = 0.66; 95 per cent c.i. 0.53 to 0.83; *P* = 0.0003).

**Fig. 5:**
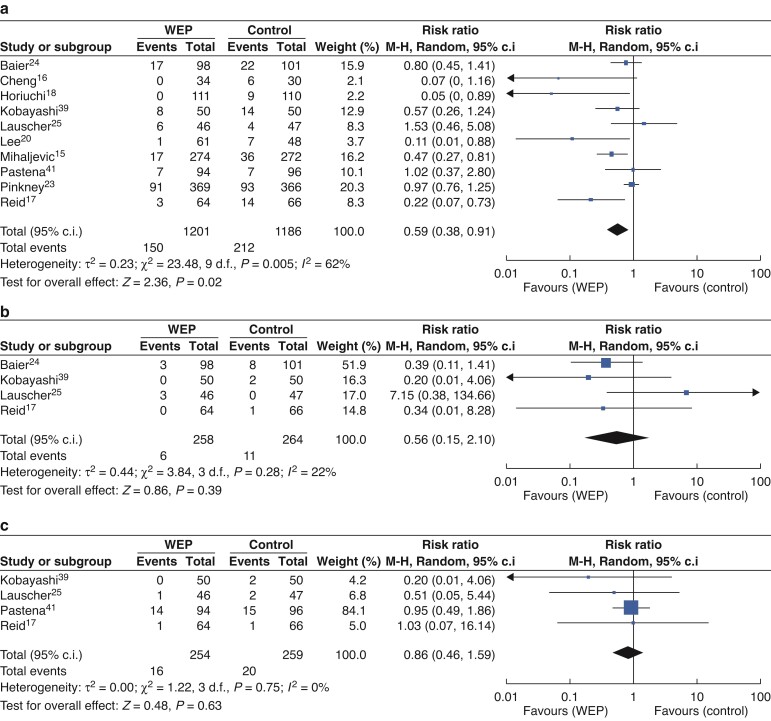
Forest plots for different degrees of SSI

The effect of WEPs in reducing different degrees of SSI (superficial, deep, and organ/space) was further analysed (*Fig. 5*). In the random-effects model, the combined RR for superficial SSI in 10 RCTs, including 2387 patients, was 0.59 (95 per cent c.i. 0.38 to 0.91; *P* = 0.02), which indicated that the incidence of SSI in the WEPs group was statistically lower. However, compared with the no WEP (NWEP) group, the WEP group has no significant differences in the incidence of deep SSI (RR = 0.56; 95 per cent c.i. 0.15 to 2.10; *P* = 0.39) and organ/space SSI (RR = 0.86; 95 per cent c.i. 0.46 to 1.59; *P* = 0.63).

**a** Superficial SSI; **b** Deep SSI; **c** Organ/space SSI; SSI, surgical site infection. WEP, wound edge protector; M-H, Mantel-Haenszel.

**Fig. 6:**
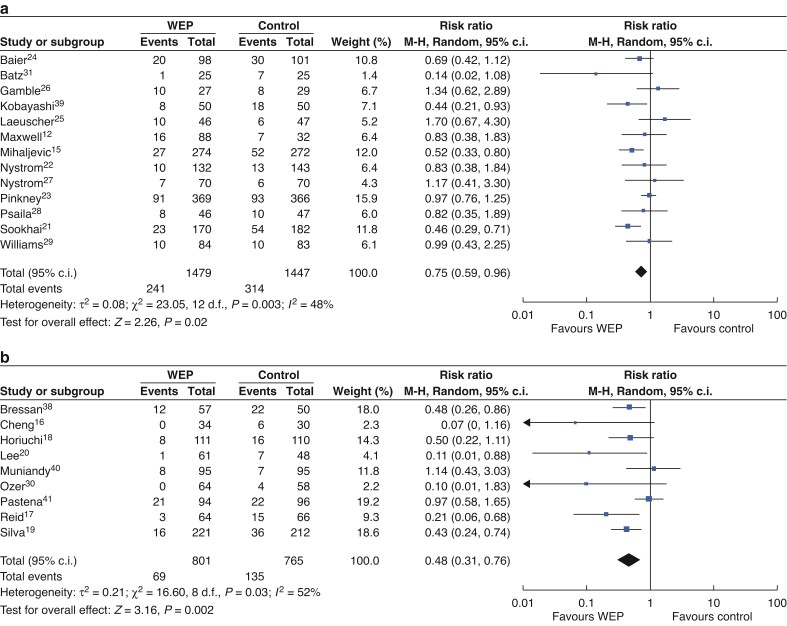
Forest plots for SSI in different types of WEPs

The effectiveness of WEPs *versus* control in different types of WEPs (single-ring or double-ring devices) was further analysed (*Fig. 6*). Thirteen RCTs involving 2926 patients were available for single-ring devices; 9 studies including 1566 patients reported data in double-ring devices. Compared with the NWEP group, there was a lower incidence of SSI in the WEP group in both subgroup analyses. The pooled RRs were 0.75 (95 per cent c.i. 0.59 to 0.96; *P* = 0.02) in single-ring devices, 0.48 (95 per cent c.i. 0.31 to 0.76; *P* = 0.002) in double-ring devices.

**a** Single-ring WEPs. **b** Dual-ring WEPs. SSI; surgical site infection; WEP, wound edge protector; M-H, Mantel-Haenszel.

### Surgical sites

**Fig. 7:**
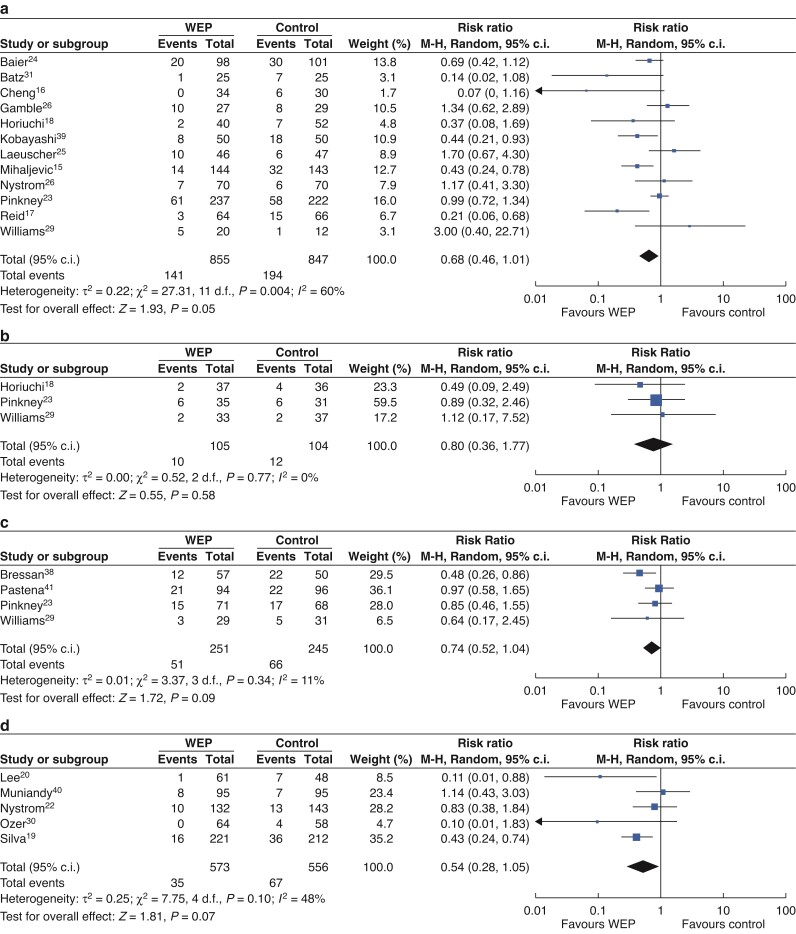
Forest plots for SSI in different abdominal surgery sites

To analyse the role of WEPs in reducing the incidence of SSI in different abdominal surgical sites, the abdominal procedures were divided into four groups: upper digestive tract/small intestine, colorectal, hepatobiliary and pancreatic (HBP), and appendix (*Fig. 7*). Twelve RCTs reported the date of 1702 patients undergoing colorectal surgery (*Fig. 7a*). The result showed the application of WEP significantly reduced the rate of SSI in the colorectal surgery subgroup (RR = 0.68; 95 per cent c.i. 0.46 to 1.01; *P* = 0.05). Three studies reported the incidence of SSI in patients undergoing upper gastrointestinal/small intestinal surgery (*Fig. 7b*). And the result was not significantly different between the two groups (RR = 0.80; 95 per cent c.i. 0.36 to 1.77; *P* = 0.58). Four studies included reported HBP cases (*Fig. 7c*). There were no significant differences in the incidence of SSI in patients undergoing HBP surgery (RR = 0.74; 95 per cent c.i. 0.54 to 1.04; *P* = 0.09). Five studies reported the incidence of SSI in patients undergoing open appendectomies (*Fig. 7d*). The pooled RRs in the random-effects meta-analysis was 0.52 (95 per cent c.i. 0.28 to 1.05; *P* = 0.07).

**a** Colorectal surgery; **b** Upper gastrointestinal/small intestinal surgery; **c** Hepatobiliary and pancreatic surgery; **d** Open appendicectomy. SSI, surgical site infection; WEP, wound edge protector; M-H, Mantel-Haenszel.

### Levels of contamination

**Fig. 8:**
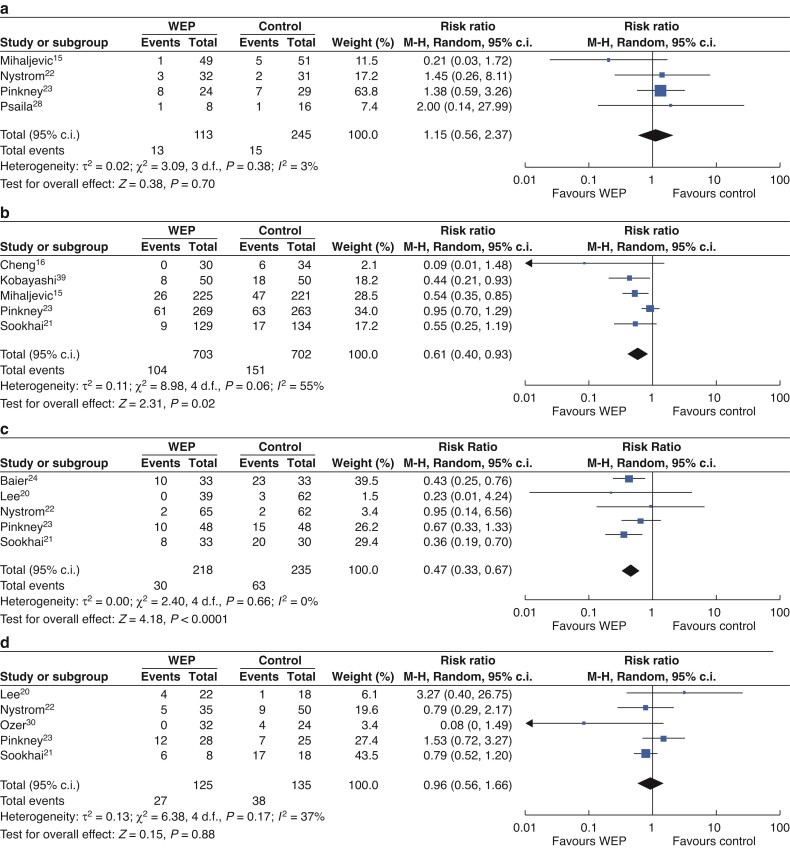
Forest plots for SSI in different degrees of contamination

Finally, the effectiveness of WEPs *versus* control in different levels of contamination (clean, clean-contaminated, contaminated, and dirty) was evaluated (*Fig. 8*). Four RCTs including 240 patients reported data in clean operations, five trials including 1405 patients in clean-contaminated surgery, five trials including 453 patients in contaminated surgery, and five trials including 260 patients reported data in dirty surgery. The pooled RRs in the random-effects meta-analysis were 1.15 (95 per cent c.i. 0.56 to 2.37; *P* = 0.70) in clean surgeries, 0.61 (95 per cent c.i. 0.40 to 0.93; *P* = 0.02) in clean-contaminated cases, 0.47 (95 per cent c.i. 0.33 to 0.67; *P* < 0.00001) in contaminated operations and 0.96 (95 per cent c.i. 0.56 to 1.64; *P* = 0.88) in dirty cases.

**a** Clean surgeries; **b** Clean-contaminated surgeries; **c** Contaminated surgeries; **d** Dirty-infected surgeries. SSI, surgical site infection; WEP, wound edge protector; M-H, Mantel-Haenszel.

## Discussion

The objectives of this review were to determine the clinical effectiveness of WEPs in reducing SSIs in patients undergoing abdominal surgery. Our systematic meta-analysis, involving 22 RCTs, and 4492 patients, demonstrated that WEP is indeed highly efficient in reducing overall SSI in patients undergoing abdominal surgery.

As WEPs were reported to be more effective for shallow SSIs, the effect of WEP on reducing different levels of SSI, grouped by the CDC classification of SSIs (superficial, deep, and organ/space) was analysed. Based on this, WEP significantly reduced superficial SSI incidence following abdominal surgery, but the effectiveness of WEP in reducing deep and organic SSIs remained unconfirmed. This may be related to the difficulty of WEP in protecting deep tissues and the abdominal cavity from infectious sources, such as intestinal content overflow.

WEP has been used to reduce SSI incidence after abdominal surgery for more than 50 years. As a result, numerous devices have been developed to serve as WEP; however, they can be divided into two major categories: single- and double-ringed. Previous studies revealed that dual-loop devices are more effective in reducing SSI incidence; however, there are limited available data on dual-ring devices, and so, sufficient robust high-quality testing is warranted^33,34^. This article includes newly published RCTs of high quality that employed dual-ring devices^38,40,41^. It has been suggested that double-ring devices might prevent SSI more effectively than single-ring devices^37^. Also a subgroup analysis herein reported is consistent with this finding; however, this is not enough to support the hypothesis, because of the huge difference in sample size (single-ring 2926 *versus* double-ring 1566), and different bias risks between the two subgroups. Based on the present results, both single-, and double-ringed WEP markedly reduced SSI incidence after abdominal surgery.

Usually, compared with other operations, colorectal surgery is associated with higher SSI rates ranging between 23 per cent and 45 per cent^43–46^. WEPs are generally reported to be more effective in clean-contaminated and colorectal operations^34^. Thus, several subgroup analyses were performed. Surprisingly, the collected data from 12 RCTs, involving 1702 patients, did not confirm a protective effect of WEPs in colorectal surgery. According to the CDC-defined wound classification, most colorectal surgeries are clean-contaminated cases, which were by far the most frequent type of surgeries conducted in the included RCTs (1405 cases out of 2358 operations that specified the degree of contamination); however, the results of different contamination levels subgroup confirmed an overall significant protective effect of WEPs in clean-contaminated surgery. The possible reason is that oral antibiotics (OABs) and mechanical bowel preparation (MBP) are gradually used in colorectal surgery. Previous evidence suggests that OAB, MBP, and their combination were associated with a significant reduction in SSI in colorectal surgery^47–49^. In addition, because of the multifactor process of SSI, changing a single factor (wound protection) may not provide significant results. Nevertheless, the subgroup analysis results of different contamination levels need to be treated with caution. This is because of the limited number of patients, high confidence intervals, and presence of high bias risk in some of the included trials.

A small number of RCTs explored WEP efficacy in reducing SSI in non-colorectal surgery. To further investigate the significance of WEP-mediated reduction of SSI at different abdominal surgical sites, three additional subgroups were evaluated: upper digestive tract/small intestine, HBP, and open appendicectomy. The WEP efficacy in reducing SSI in those surgery remains unknown. This is likely due to the multifactorial process in which SSI occurs. Therefore, altering a single factor (wound protection) may not provide significant results; however, as the subgroup analyses here reported, contained a limited number of patients, more high-quality RCTs are needed to reach a reliable conclusion.

The results of this updated review are similar to the findings of recently published systematic reviews. A past study included six RCTs and it revealed that wound protectors markedly reduced rates of SSI after gastrointestinal and biliary surgery^37^. In addition, 2 meta-analyses^32,36^, involving 11, and 12 RCTs respectively, showed that WEPs significantly lowered SSI incidence in patients undergoing laparotomies. Another systematic review performed subgroup analyses on single- *versus* double-ringed WEP, varying wound contamination levels, and varying SSI depths during colorectal surgery, and their results were also similar to those presented in this study^34^. Likewise, the results of another study, involving four small, randomized trials, showed beneficial effects of WEP in reducing SSI after open appendectomy^50^. The largest previous meta-analysis^35^ and the latest systematic review and meta-analysis^33^ evaluating the efficacy of a WEP in abdominal surgery, included 18 RCTs and 14 RCTs respectively, and confirmed that a WEP can significantly reduce SSI incidence after laparotomy.

This study has few limitations. First, heterogeneity bias was inevitable in the process of study inclusion criteria, SSI definition, control intervention, inter-study follow-up interval, and long-term implementation of included trials. Second, it was not possible to unify WEPs in terms of manufacturing materials, shapes or forms, and specific functions. The increased application of WEPs over time may have influenced differences in results between studies conducted at different times. In addition, although both electronic, and manual searches to identify potentially relevant articles were conducted, some meaningful articles could be missed, especially those that were not published in English. Finally, the results of some subgroup analyses must be treated with caution due to the insufficient and relatively poor-quality data in the included literature. Therefore, there is a great need for a more rational design and rigorous implementation of large-scale multicenter RCTs, particularly concerning WEP’s efficacy in preventing SSI in patients undergoing abdominal surgery with different abdominal surgical sites, different surgical modalities, and different degrees of contamination.

## Supplementary Material

zrac065_Supplementary_Data

## Data Availability

The datasets during the present study are available from the corresponding author on reasonable request.
